# Acanthamoeba Keratitis After Laser-Assisted In Situ Keratomileusis (LASIK) Successfully Treated With Polyhexamethylene Biguanide (PHMB) 0.08% Monotherapy: A Case Report

**DOI:** 10.7759/cureus.108725

**Published:** 2026-05-12

**Authors:** Diya Baker, Kanellina Kanellopoulou, Nick Kopsachilis, Lanxing Fu

**Affiliations:** 1 Ophthalmology, Sandwell and West Birmingham Hospitals NHS Trust, Birmingham, GBR; 2 Ophthalmology, East Kent Hospitals University NHS Foundation Trust, Canterbury, GBR

**Keywords:** acanthamoeba keratitis (ak), confocal microscopy, laser-assisted in situ keratomileusis, polyhexamethylene biguanide, refractive surgery complications

## Abstract

A man in his early 30s presented with progressive unilateral ocular pain and visual deterioration three months after uncomplicated bilateral laser-assisted in situ keratomileusis (LASIK). Initial treatment for presumed bacterial keratitis, followed by herpes simplex keratitis, failed to result in improvement. He was referred to our corneal service, where in vivo confocal microscopy demonstrated characteristic *Acanthamoeba* cysts within the corneal stroma. The patient was commenced on polyhexamethylene biguanide (PHMB) 0.08% monotherapy through an Early Access Programme in accordance with the Orphan Drug for Acanthamoeba Keratitis protocol, with subsequent clinical and symptomatic improvement. Resolution was achieved without the use of topical corticosteroids. This case highlights the diagnostic difficulty of *Acanthamoeba* keratitis in post-refractive surgery patients without a history of contact lens use and emphasises the importance of early clinical suspicion, advanced diagnostics, and protocol-based therapy to optimise outcomes.

## Introduction

*Acanthamoeba* keratitis (AK) is a rare but potentially sight-threatening corneal infection caused by free-living protozoa found in soil, freshwater, and air. Although most commonly associated with contact lens wear, a proportion of cases occur in non-contact lens users, often following corneal trauma or surgery. Risk factors include environmental exposure and epithelial compromise [1,2].

Laser-assisted in situ keratomileusis (LASIK) is widely performed and associated with a low incidence of infectious complications. However, the procedure results in long-term alterations in corneal architecture, including reduced corneal nerve density, changes in keratocyte populations, and altered local immune responses that may impair epithelial barrier function and delay pathogen recognition [3].

AK following LASIK is uncommon and may be difficult to diagnose because early clinical features overlap with bacterial or herpetic keratitis. Delayed diagnosis is associated with poorer visual outcomes and prolonged treatment [2]. Reports of post-LASIK AK in non-contact lens users treated with contemporary polyhexamethylene biguanide (PHMB) 0.08% monotherapy remain limited.

## Case presentation

A man in his 30s presented with a one-month history of worsening ocular pain (Table 1), photophobia, redness, and blurred vision in the right eye. He worked as a gardener with regular exposure to soil, organic material, and environmental water sources. Three months earlier, he had undergone uncomplicated bilateral LASIK for myopia, achieving unaided visual acuity of 6/5 in both eyes.

**Table 1:** Clinical timeline of key events. LASIK: laser-assisted in situ keratomileusis; PHMB: polyhexamethylene biguanide

Time	Event
Month 0	Bilateral LASIK
Month 2	Onset of pain and visual symptoms
Month 3	Treated empirically as bacterial keratitis
Month 3.5	Treated as herpes simplex keratitis
Month 4	Referral; confocal microscopy confirmed *Acanthamoeba*
Month 4	PHMB 0.08% initiated
Month 10	Complete clinical resolution and cessation of PHMB 0.08%
Month 16	Asymptomatic unaided vision 6/9.5, pinhole 6/7.5

He denied contact lens use and reported no definite ocular trauma, although he frequently worked outdoors without protective eyewear. His medical history was unremarkable.

Initial assessment elsewhere identified a corneal epithelial defect without stromal infiltrate or anterior chamber reaction, and empirical treatment with topical chloramphenicol and lubricants was initiated. Symptoms partially improved but subsequently worsened. On re-presentation, pain was severe and disproportionate to clinical findings, and visual acuity had deteriorated to counting fingers. Slit-lamp examination revealed a paracentral epithelial defect, stromal infiltrates, radial perineuritis, and marked ciliary injection. The fellow eye was unaffected.

Herpes simplex keratitis was suspected due to reduced corneal sensation, and antiviral therapy was commenced without improvement, prompting referral to a tertiary corneal service.

**Figure 1:**
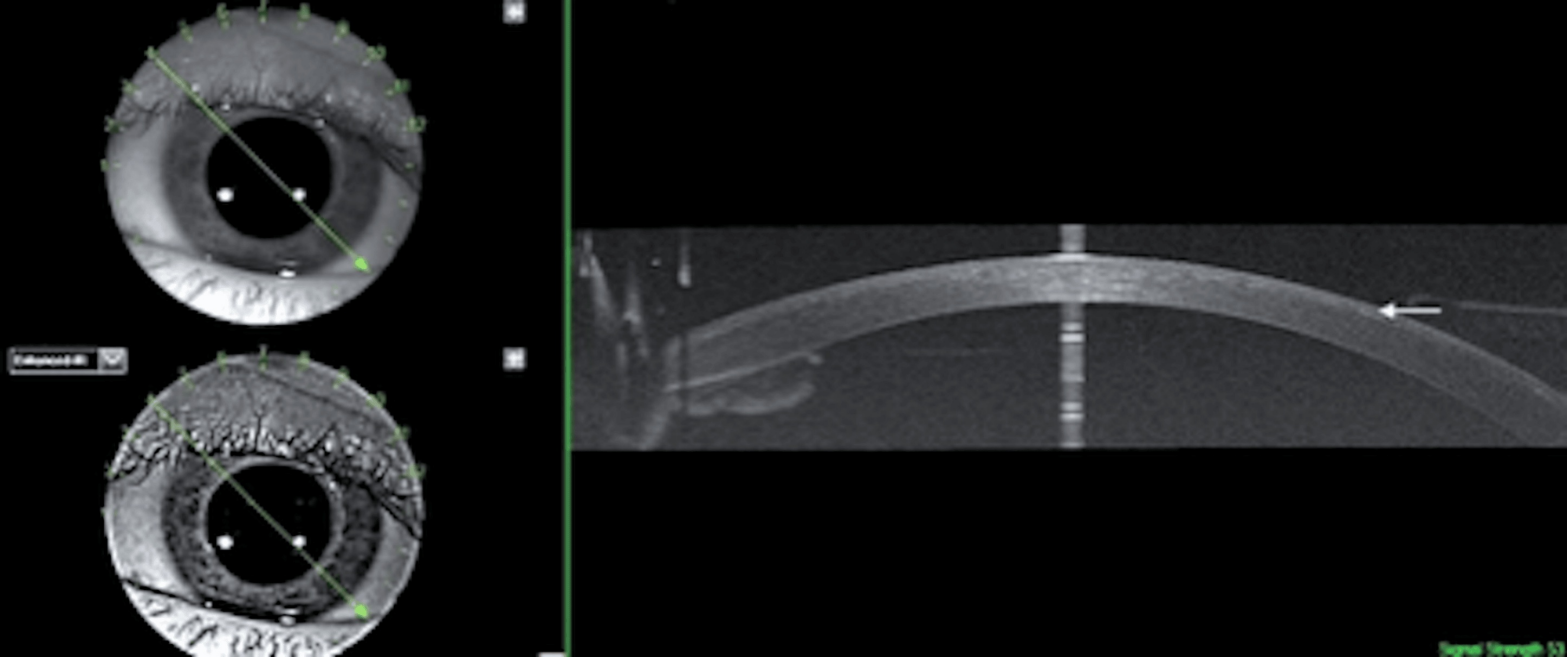
Anterior segment optical coherence tomography (AS-OCT) demonstrating increased laser-assisted in situ keratomileusis (LASIK) flap interface reflectivity at month 4. The arrow indicates the increased reflectivity at the LASIK flap interface following infection, as visualized on anterior segment OCT.

Anterior segment optical coherence tomography demonstrated stromal involvement beneath the epithelial defect (Figure 1). In vivo confocal microscopy revealed numerous hyper-reflective, double-walled cystic structures within the corneal stroma, consistent with *Acanthamoeba*. Corneal scraping was not performed due to characteristic confocal findings and the need for prompt treatment initiation.

**Figure 2:**
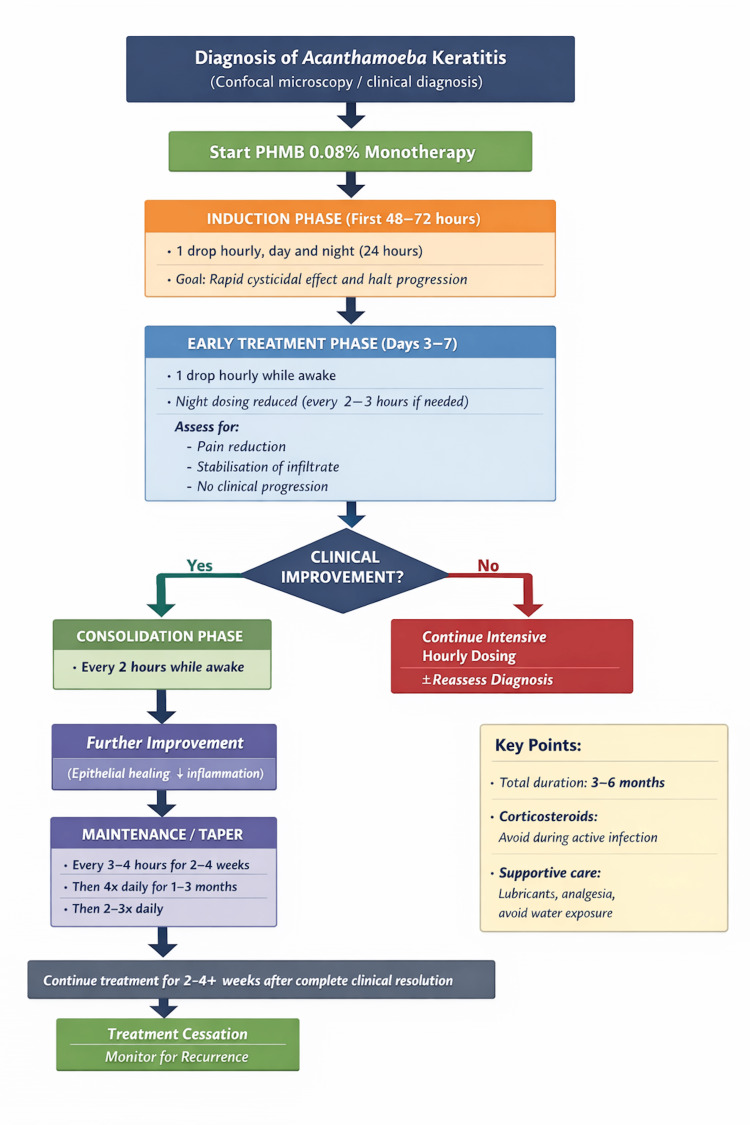
The polyhexamethylene biguanide (PHMB) 0.08% monotherapy treatment schedule for the patient according to the Orphan Drug for Acanthamoeba Keratitis (ODAK) protocol.

The patient was commenced on PHMB 0.08% (AKANTIOR®, SIFI, Italy) ophthalmic solution as monotherapy via an Early Access Programme in accordance with the Orphan Drug for Acanthamoeba Keratitis (ODAK) protocol (Figure 2). Drops were administered hourly initially and gradually tapered according to clinical response. Topical corticosteroids were withheld.

**Figure 3:**
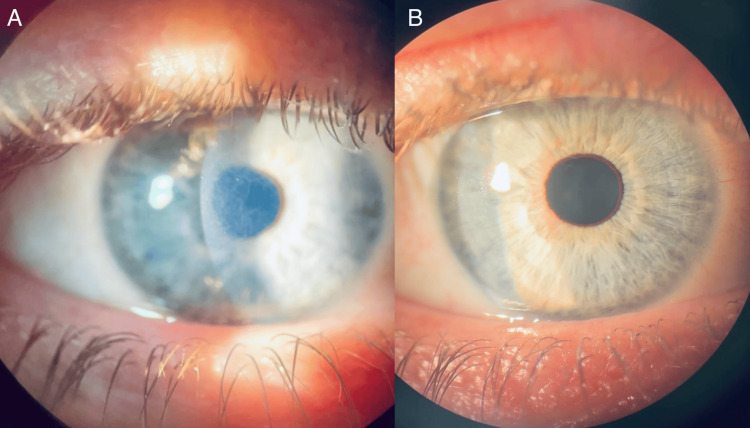
Serial anterior segment photographs showing clinical resolution of Acanthamoeba keratitis. Panel A demonstrates the early interface haze one month after initiation of polyhexamethylene biguanide 0.08%, and panel B shows the clinical resolution at month 16 with best-corrected visual acuity of 6/7.5.

Supportive treatment included preservative-free lubricants, oral analgesia, avoidance of water exposure, and advice regarding protective eyewear. Over subsequent weeks, symptoms improved. Serial examinations demonstrated epithelial healing, reduction in stromal haze (Figure 3), and resolution of perineuritis. Treatment was tapered according to protocol, with no recurrence. Six months after cessation of PHMB 0.08%, unaided visual acuity was 6/9.5, improving to 6/7.5 with pinhole, and the infection had completely resolved.

## Discussion

AK should be considered in patients with atypical or progressive keratitis following refractive surgery, even in the absence of contact lens use. Disproportionate pain and failure to respond to standard antimicrobial or antiviral therapy should prompt early reconsideration of the diagnosis. In vivo confocal microscopy enables rapid, non-invasive confirmation. Post-LASIK infectious keratitis may be bacterial, fungal, viral, or protozoal [2]. Environmental exposure, epithelial compromise, and altered corneal innervation may increase susceptibility even without contact lens use [1].

Occupational and environmental risk factors should be considered when selecting refractive procedures. Compared with LASIK, surface ablation (photorefractive keratectomy; PRK) and small incision lenticule extraction (SMILE) preserve anterior corneal integrity and are associated with fewer epithelial defects and less disruption of corneal innervation, potentially reducing infection risk [3,4]. Topical corticosteroids were withheld due to concerns regarding enhancement of amoebic proliferation and a lack of consensus on their use during active infection [5].

The Phase III ODAK trial demonstrated that PHMB 0.08% monotherapy is non-inferior to combination therapy and supports a standardised treatment approach [6,7]. Protocol-driven management and early diagnosis using confocal microscopy may reduce morbidity and the need for surgical intervention [8-10].

## Conclusions

AK should be considered in patients presenting with atypical or progressive keratitis following refractive surgery, even in the absence of contact lens use. Disproportionate pain and failure to respond to conventional antimicrobial or antiviral therapy should prompt early reconsideration of the diagnosis. In vivo confocal microscopy enables rapid confirmation, and early initiation of protocol-based PHMB 0.08% monotherapy can result in clinical resolution and good visual recovery without the need for topical corticosteroids. This case also highlights the need for occupational risk assessment in refractive surgery candidates. Early confocal imaging and standardised therapy may improve outcomes in atypical post-refractive surgery keratitis.
